# Diagnostic Value of Systematic Imaging Examination in Embedded Optic Disc Drusen in Adolescents with Mild Visual Impairment

**DOI:** 10.1155/2020/6973587

**Published:** 2020-04-03

**Authors:** Xiuhua Jia, Tiancheng Bao, Shasha Wang, Ting Jiang, Zhijian Zhong, Yanling Zhang, Qigen Li, Xiang Zhu

**Affiliations:** ^1^Department of Ophthalmology, Third Affiliated Hospital of Sun Yat-Sen University, 600# Tianhe Road, Guangzhou, Guangdong Province 510630, China; ^2^Department of Radiology, Third Affiliated Hospital of Sun Yat-Sen University, 600# Tianhe Road, Guangzhou, Guangdong Province 510630, China; ^3^Department of Ultrasonography, Third Affiliated Hospital of Sun Yat-Sen University, 600# Tianhe Road, Guangzhou, Guangdong Province 510630, China; ^4^Department of Infectious Diseases, Third Affiliated Hospital of Sun Yat-Sen University, 600# Tianhe Road, Guangzhou, Guangdong Province 510630, China

## Abstract

**Aim:**

To evaluate the diagnostic value of systematic ophthalmologic imaging examination in the diagnosis of embedded optic disc drusen (ODD) in adolescents with mild visual impairment.

**Methods:**

Eleven patients were evaluated through optometric examination, fundus photography, visual field inspection, optical coherence tomography (OCT), ultrasonography (US), and fundus fluorescein angiography (FFA). Of the 11 patients, three also underwent cranial and orbital magnetic resonance imaging (MRI).

**Results:**

All 11 patients had either no apparent abnormality or only mild refractive abnormalities. In all patients, fundus inspection revealed flushing the optic disc with varying degrees of limited boundary ambiguity and optic disc congestion with disappearance of the fovea. One patient had a visual field defect during the period of edema of ODD, but the visual field returned to normal after the optic disc edema subsided. US revealed discoid acousto-optic masses in front of the optic disc in six patients. OCT showed a slight elevation and thinning of the retinal nerve fiber layer (RNFL) of the optic disc in all patients. Quasicircular, hyperreflex signals of different sizes could be observed below the RNFL. Late-stage FFA revealed focal staining at the edge of the optic disc without fluorescence leakage in all patients. Orbital and cranial MRI findings were normal in the three patients.

**Conclusion:**

A systematic ophthalmologic imaging examination can not only improve the detection rate of embedded ODD but also avoid excessive examinations and treatments.

## 1. Introduction

Optic disc drusen (ODD) are deposits of vitreous material on the optic disc, which lead to swelling of the disc, different amounts of bleeding around the disc, or even secondary optic atrophy. This disease, which can develop in one or both eyes, can cause varying degrees of visual impairment. Most ODD are embedded in the optic disc. Early in the disease process, the uncalcified ODD are usually buried in the optic nerve head tissue and may cause few clinical symptoms. As the visual function of the patient is rarely affected, this condition is often neglected during clinical examination. With increases in ODD volume and degree of calcification, the fundus of adolescent patients is characterized by flushing, crowding, and blurred visual disc halo, which can be easily detected using B-mode ultrasonography. With the application of optical coherence tomography (OCT) in high-resolution imaging, even smaller and deeper ODD can be presently detected. Nevertheless, the diagnosis of ODD in adolescent patients with mild visual impairment accompanied by the optic disc morphology mentioned above has not attracted much attention from clinicians. Since there was no generally accepted definition of mild visual impairment, in our study, we defined mild visual impairment as a visual acuity of the affected eye between 0.6 and 0.9. Although it is often considered a benign disease, most patients with ODD are not diagnosed until there is visual field defect, sudden visual loss, optic disc hemorrhage, or even optic disc neovascularization. Patients with early ODD are often diagnosed with papillary edema caused by papillitis or increased intracranial pressure due to the peculiar shape of the optic disc. Thus, they usually undergo neurological examination and treatment. Despite advances in ophthalmology, imaging modalities, if used in isolation, are more likely to lead to misdiagnosis of ODD [1]. Moreover, the diagnosis rate of minor ODD in adolescents with mild visual impairment and no fundus hemorrhage remains low. This may lead to missed diagnoses, misdiagnosis, and overtreatment. A previous study showed that it is more cost-effective to perform ophthalmologic ultrasonography prior to neuroimaging, especially when the patient is asymptomatic [2, 3]. Thus, systematic ophthalmologic examination is particularly important in the diagnosis of embedded ODD in adolescents with mild visual impairment.

We evaluated 11 adolescents with mild visual impairment accompanied by disc flushing, crowded optic disc, and blurred disc halo and attempted to determine the cause of misdiagnosis and missed diagnosis through fundus photography, OCT, B-mode ultrasonography, and fundus fluorescein angiography (FFA). The standard diagnostic scheme of juvenile patients with embedded ODD was evaluated.

## 2. Subjects and Methods

### 2.1. General Information

This is a retrospective study, and all procedures performed in studies involving human participants were in accordance with the ethical standards of the Ethics Committee of the Third Affiliated Hospital of Sun Yat-Sen University for Human Study (registration number, NCT03957642) and the 1964 Declaration of Helsinki and its later amendments or comparable ethical standards. Informed consent was obtained from all individual participants included in the study. Between January 2016 and December 2017, 11 patients (2 men and 9 women; age range, 13–23 years) with mild visual impairment with different degrees of local boundary ambiguity in their optic discs and optic disc congestion were enrolled in the study. The initial diagnosis of these patients was visual impairment of unknown origin. All patients denied a family history of any related disease.

### 2.2. Methods

All patients underwent optometric examination, intraocular pressure measurement, slit-lamp examination, B-mode ultrasonography (Mindray Resona 7), visual field inspection (Carl Zeiss HFA740i), FFA (Carl Zeiss FF450), fundus color photography, and spectral domain (SD)-OCT (Topcon 3D OCT-2000).

We used the standard logarithmic visual acuity chart, which used an “E” notion, in performing the visual acuity test. We used SD-OCT (wavelength 840 nm) to detect the depth of ODD and measure the shape of the optic disc and thickness of the retinal nerve fiber layer (RNFL) around it. The central 30-2 threshold test and SITA Fast strategy were used in the automatic analysis of the visual field.

## 3. Results

All 11 patients were diagnosed with embedded ODD, including 5 cases of binocular embedded ODD and 6 cases of monocular embedded ODD.

### 3.1. Visual Examination

The first patient was initially diagnosed with optic papillitis and treated with hormone shock therapy. Following neurological consultation, lumbar puncture and orbital and cranial magnetic resonance imaging (MRI) were performed. Of the remaining ten patients, only two underwent orbital and cranial MRI. The average follow-up duration was 1 year after the first visit; no treatment was provided during the follow-up period. All patients had mild visual impairment at the first visit. Optometric examination revealed no ametropia or only mild myopia in these patients ([Supplementary-material supplementary-material-1]).

### 3.2. Fundus Photography and FFA

**Figure 1:**
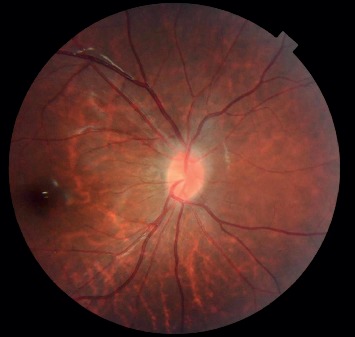
Fundus photography findings of a patient with ODD. Local flushing of the optic disc, congestion of the disc with disappearance of the fovea, and unclear boundary of the disc were observed.

**Figure 2:**
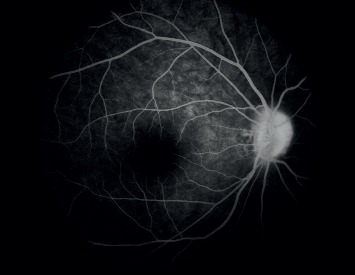
FFA of a patient with ODD. FFA reveals a slightly blurred optic disc boundary. No obvious fluorescence leakage was observed during the prolongation of imaging time. However, in the late stage, light fluorescence staining was noted at the edge of the local optic disc.

Local flushing of the optic disc, congestion of the disc with disappearance of the fovea, and unclear boundary of the disc were observed in all 11 (100%) patients (Figure 1). FFA findings revealed slight blurring of the optic disc boundary in all patients. No obvious fluorescence leakage was observed during the prolongation of the imaging time; however, in the late stage, light fluorescent staining could be observed at the edge of the local optic disc (Figure 2).

Especially, the diagnosis of monocular ODD (discoid strong echoes in the optic disc only in one eye) based on the B-mode ultrasonography was not supported by subsequent FFA, which suggested the presence of binocular ODD.

### 3.3. B-Mode Ultrasonography

**Figure 3:**
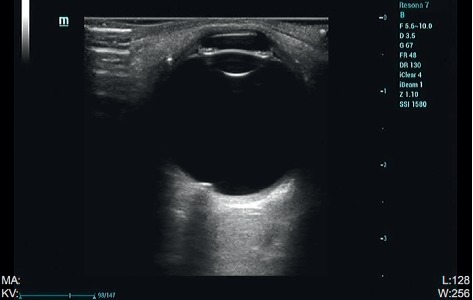
B-mode ultrasonography findings of a patient with ODD. B-mode ultrasonography shows discoid strong echoes with irregular flat protuberances in the optic disc.

B-mode ultrasonography findings revealed discoid strong echoes with irregular flat protuberances in the optic disc in six (54.5%) patients. No abnormal echoes were found in the other five (45.5%) patients (Figure 3).

### 3.4. SD-OCT

**Figure 4:**
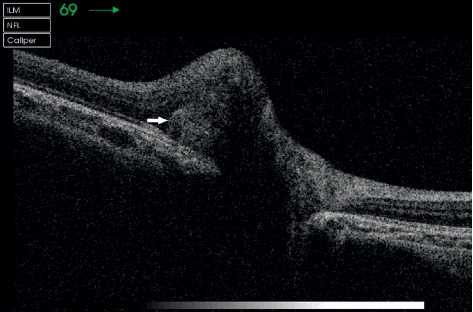
OCT of a patient with ODD. High-reflex dense points with different volumes of quasicircular shadows can be observed in the optic papillary nerve fiber layer. The white arrow indicates ODD.

SD-OCT was used to examine the optic disc by section and volume scan. The results revealed high-reflex dense points with different volumes of quasicircular shadows in the optic papillary nerve fiber layer. These results were observed in all 11 patients (Figure 4). There was only one patient with obvious optic disc edema. We found that, during the period of optic disc edema, the thicknesses of the RNFL on the upper, lower, nasal, and temporal sides of the optic disc were 170 *μ*m, 174 *μ*m, 123 *μ*m, and 136 *μ*m, respectively. After the edema subsided, the thicknesses of the RNFL on the upper, lower, nasal, and temporal sides of the optic disc returned to normal, which were 118 *μ*m, 124 *μ*m, 65 *μ*m, and 74 *μ*m, respectively.

### 3.5. Visual Field Inspection

Only one patient presented with an abnormal visual field. This finding was observed during the period of edema of ODD, but the visual field returned to normal after the optic disc edema subsided ([Supplementary-material supplementary-material-1]). With the development of edema of the optic disc, the visual field inspection of this patient showed an enlargement of the physiological blind spot. The physiological blind spot of the patient can gradually return to normal when the edema of optic disc subsided.

## 4. Discussion

The prevalence of ODD is approximately 2.4% [4]; however, its etiology and pathogenesis remain unclear. ODD usually develop in patients with a small, crowded optic disc. Children with small scleral sieve plate diameters are more likely to have the disease. The death of ganglion cell axons caused by the accumulation of axoplasm, as a result of congenital micro-optic disc and scleral sieve plate stenosis, is the likely pathogenesis of the disease [5]. In its early stage, uncalcified ODD are usually buried in the optic nerve head tissue and cause few clinical symptoms. As the visual function of patients is rarely affected, this condition is often neglected during routine clinical examination. However, the size and location of ODD change with age, and the squeezing effect of ODD on the surrounding tissues of the optic disc increases with increasing age of the patient. With the increase in the size of and calcium deposition in ODD, visual field defect symptoms of the patient worsen [6]. The disease usually results in only mild visual impairment and rarely causes visual loss. However, in the later stages of the disease, subretinal hemorrhage and anterior ischemic optic neuropathy may develop due to direct mechanical compression of ODD on the optic disc. Additionally, mechanical damage due to ODD around the optic papilla and release of vascular endothelial growth factor may lead to choroidal neovascularization (CNV) and/or polypoid choroidal vasculopathy with secondary subretinal hemorrhage, which may cause significant visual impairment. Moreover, a family history of glaucoma was significantly more frequent in patients with ODD with an incidence of 20.7% [7]. Small and crowded discs in patients with ODD often affect the optic cup enlargement phenomenon of glaucoma and may even lead to errors in determining the ultimate visual field damage due to glaucoma or ODD [8]. These findings also indicate a dangerous progression of the disease. Therefore, detecting early embedded ODD in children and adolescents and improving the diagnostic rate of the disease have great clinical value.

Vision decline is a common clinical finding in adolescents. Eye development is in a growth period till the age of 14 years. This phenomenon highlights the importance of paying attention to ametropia in adolescent patients with poor vision. We found that the onset of ODD was insidious and the symptoms were mild because early mild embedded ODD was deeply buried in the optic disc and covered by optic nerve fibers. The patients in this study presented with only mild visual impairment and some even had normal vision and visual fields.

Fundus photography findings of patients with ODD showed flushing of the optic disc, congestion of the disc with disappearance of the fovea, and unclear boundary of the local disc, which are similar to the findings in hyperopia. After refractive correction, the visual acuity of these patients is often unsatisfactory. When the defects in visual acuity cannot be explained based on the refractive state, the defect may easily be misdiagnosed as amblyopia or even neurological disease. As a result of misdiagnosis, patients may be transferred to the neurology department and undergo inappropriate examination and treatment. Ophthalmologists need to consider ODD in patients with mild visual impairment. Systematic ophthalmologic imaging is essential in improving the diagnostic rate of embedded ODD in adolescents with mild visual impairment.

All our patients had poor vision correction and were evaluated through optometry, intraocular pressure examination, slit-lamp examination, B-mode ultrasonography, visual field inspection, FFA, fundus photography, and SD-OCT. One patient had also undergone neurological examination and treatment, and three patients had undergone orbital and cranial MRI. Embedded ODD with different degrees of calcification, volumes, and depths can cause varying visual field defects. Individuals with mild symptoms do not usually have any visual field abnormalities, while those with severe symptoms might have visual field defects related to physiological blind spots of varying degrees. B-mode ultrasonography is a fast, reliable, economical, and effective method for the diagnosis of ODD. It can help detect clear echoes of ODD with calcification. Even deeply calcified ODDs can be detected [9–11]. However, the resolution of B-mode ultrasonography is low, and the obtained image is closely related to the location, size, and composition of ODD. Thus, locating ODD through B-mode ultrasonography is difficult when it is deep and small and has low calcium content. Furthermore, B-mode ultrasonography does not reveal the degree of damage to the RNFL. Embedded ODD, which has not been calcified, is difficult to recognize through fundus photography, B-mode ultrasonography, or computed tomography. OCT can help distinguish the subtle changes in retinal structure through high-resolution imaging and tracking positioning technology and detecting size and location changes in ODD. OCT can help locate some of the buried ODDs that cannot be detected through B-mode ultrasonography; thus, it provides a precise basis for diagnosis and helps avoid overtreatment. OCT reveals a circular reflection signal under the optic disc nerve fiber layer and between the nerve epithelial layers. The edge of the signal is high, and the center of the signal is low [12–14]. Moreover, thinning of the nerve fiber layer can be found on OCT [15]. The optic cup corresponding to the signal is shallow. The mass hyperreflex signal in patients with optic disc edema is weakened, and the boundary is slightly blurred. Thus, OCT is extremely helpful in differentiating optic disc edema from pseudoedema, optic nerve head drusen, and other optic neuropathies [15–17]. However, OCT also has limitations. A small uncalcified ODD or bilateral ODD with a small volume may be easily missed. Ophthalmologists without much experience may even identify the flushed and crowded optic discs as a normal developmental congenital morphological abnormality. Presently, by combining OCT and FFA, the detection rate of small-scale ODDs can be improved. FFA of a buried ODD shows strong local fluorescence, but there was no dye leakage in the location of the buried ODD in the late stage. In our first three cases, MRI findings did not reveal any abnormality, but ODD was detected in these cases through subsequent systematic ophthalmologic examination.

Thus, it is crucial to develop a systematic and standardized protocol for eye examination of adolescents presenting with mild visual impairment accompanied by flushing of the optic papilla and congestion of the optic disc; it may help avoid unnecessary treatment and surgery. The following criteria should be considered when screening adolescents with the abovementioned conditions in ODD: (1) the patient has an unexplained visual impairment with or without visual field defect due to the abnormal shape of the optic papilla; (2) on OCT, different volumes of quasicircular echoes are found that appear to be encapsulated, with high margins and low central reflections; (3) B-mode ultrasonography reveals disc calcification echoes; (4) FFA findings reveal focal strong fluorescence at the edge of the optic disc in the late stage. B-mode ultrasonography is of great importance in the diagnosis of ODD. It is a noninvasive modality, whereas FFA is an invasive procedure. Some patients may experience anaphylaxis or even anaphylactic shock following the injection of the contrast medium. Particularly, patients with impaired renal function are not eligible to undergo FFA. Additionally, in adolescent patients with visual impairment, it is necessary to distinguish ODD from edema of the optic papilla. Routine ophthalmologic examinations, especially B-mode ultrasonography and OCT, should be initially considered, as opposed to the head and orbital MRIs. The abovementioned scheme can not only greatly improve the detection rate of ODD but also reduce the burden of unnecessary examinations for patients. However, it is imperative to avoid ignoring a true neurological disease.

ODD can cause serious visual field defects and vascular complications, such as CNV [18, 19]. Therefore, regular examinations of the visual field, intraocular pressure, and nerve fiber layer should be performed in patients with ODD after diagnosis. A systematic process for taking advantage of the various types of ophthalmologic imaging modalities in patients with low vision will help improve the detection rate of mild ODD, avoid complications of the disease, and prevent the persistent decline in the patients' vision. We also found that, in a few cases, ODD and increased intracranial pressure can coexist and that a diagnosis of drusen does not preclude the need for investigations for papilledema. Systematic ophthalmologic imaging can greatly reduce the harm caused by the disease to adolescent patients and improve their quality of life.

This study also has limitations, as the sample size is small and a prospective study may be helpful in improving our conclusion.

## Data Availability

The data used to support the findings of this study are included within the article.

## Supplementary Materials

Supplementary Materials Table 1: systematic ophthalmologic examinations of the 11 patients.
