# Supplementary material for: Diagnostic Value of Systematic Imaging Examination in Embedded Optic Disc Drusen in Adolescents with Mild Visual Impairment
Source: J Ophthalmol. 2020 Apr 3;2020:6973587. doi: 10.1155/2020/6973587 (PMC7165333; doi:10.1155/2020/6973587)
Supplement: Supplementary Materials — Table 1: systematic ophthalmologic examinations of the 11 patients. [file 6973587.f1.docx]

|  | **Sex** | **Age** | **Monocular/**  **Binocular ODD** | **Corrected visual acuity** | **Refractive status** | **Visual field** | **B-ultrasonography** | **OCT** | **FFA** | **Preliminary diagnosis** |
| --- | --- | --- | --- | --- | --- | --- | --- | --- | --- | --- |
| 1 | F | 14 | Monocular | OD: 0.6  OS: 1.2 | OD: −O.75DS  OS: 0.50DC × 75 | Physiological  blind spot enlargement | ODD (+) | ODD (+) | ODD (+) | Suspicious optic papillitis |
| 2 | F | 23 | Binocular | OD: 0.9  OS: 0.9 | OD: −2.75DS/0.50DC × 120  OS: −2.50DS/0.50DC × 100 | Normal | ODD (+) | ODD (+) | ODD (+) | Ametropia |
| 3 | M | 21 | Binocular | OD: 0.8  OS: 0.8 | OD: −0.75DS/0.25DC × 75  OS: −0.50DS/0.50DC × 45 | Normal | ODD (+) | ODD (+) | ODD (+) | Ametropia |
| 4 | M | 17 | Binocular | OD: 0.8  OS: 0.9 | N | Normal | ODD (+) | ODD (+) | ODD (+) | Suspicious optic papillitis |
| 5 | F | 22 | Monocular | OD: 0.8  OS: 1.0 | OD: −0.25DS/0.50DC × 95  OS: −0.50DS/0.50DC × 115 | Normal | ODD (−) | ODD (+) | ODD (+) | Suspicious optic papillitis |
| 6 | F | 13 | Binocular | 0.8 | N | Normal | ODD (−) | ODD (+) | ODD (+) | Suspicious optic papillitis |
| 7 | F | 20 | Monocular | 0.8 | OD: −2.00DS  OS: −3.00/0.50DC × 15 | Normal | ODD (−) | ODD (+) | ODD (+) | Ametropia |
| 8 | F | 18 | Monocular | 0.6 | N | Normal | ODD (−) | ODD (+) | ODD (+) | Suspicious optic papillitis |
| 9 | F | 19 | Monocular | 0.8 | N | Normal | ODD (−) | ODD (+) | ODD (+) | Suspicious optic papillitis |
| 10 | F | 21 | Binocular | 0.8 | OD: −1.75DS  OS: −2.00DS/0.75DC × 117 | Normal | ODD (+) | ODD (+) | ODD (+) | Ametropia |
| 11 | F | 17 | Binocular | O.8 | OD: −0.75DS/0.25DC × 180  OS: −1.50DS | Normal | ODD (+) | ODD (+) | ODD (+) | Suspicious optic papillitis |

**Table 1**. Systematic ophthalmologic examinations of the 11 patients
